# Arterial Stiffness May Predict Subsequent Cancer Therapy-Related Cardiac Dysfunction in Breast Cancer Patients

**DOI:** 10.1007/s12012-024-09841-w

**Published:** 2024-03-08

**Authors:** Mürsel Şahin, Seher Nazlı Kazaz, Fatih Kartaler, Burcu Kodal, Seda Altuntaş, Elif Yüce, Oğuzhan Ekrem Turan, Merih Kutlu

**Affiliations:** 1 Department of Cardiology, Faculty of Medicine, Karadeniz Technical University, 61000 Trabzon, Turkey; 2 Department of Medical Oncology, Faculty of Medicine, Karadeniz Technical University, Trabzon, Turkey; 3 Soma State Hospital, Manisa, Turkey; 4 Department of Medical Oncology, Karaman Training and Research Hospital, Karaman, Turkey; 5 Department of Cardiology, Faculty of Medicine, Dokuz Eylul University, İzmir, Turkey

**Keywords:** Breast cancer, Arterial stiffness, Cancer therapy-related cardiac dysfunction, Global longitudinal strain

## Abstract

Cancer therapy-related cardiac dysfunction (CTRCD) is still a serious problem. Existing risk scores are insufficient for risk classification, especially in low and medium-risk patients. This study aims to evaluate if arterial stiffness (AS) measurement, which is associated with most of the known risk factors, can be a useful parameter for predicting subsequent CTRCD in patients with breast cancer (BC). Patients with BC were included in the study. All patients’ AS parameters such as pulse wave velocity (PWV), augmentation index (AIx), augmentation pressure (AP), and echocardiographic parameters were obtained before treatment. During treatment, echocardiographic follow-up with routine parameters and left ventricle global longitudinal strain (LVGLS) were measured. Patients were evaluated on whether CTRCD occurred or not. A total of 67 patients were analyzed. The mean age of the study population was 54.9 ± 11 years. Baseline characteristics were similar except for age. No CTRCD diagnosis was obtained according to left ventricle ejection fraction (LVEF) reduction, but 18 patients (26.8%) developed CTRCD regarding the decline in LVGLS. Left ventricle hypertrophy and diastolic dysfunction were more frequent in patients with CTRCD (p = 0.016 and p = 0.015, respectively). PWV, AIx, and AP as AS parameters were significantly higher in patients with CTRCD, but Alx@75 were not (p = 0.005, p = 0.034, p = 0.008, p = 0.077, respectively). A positive correlation between PWV and a decreased percent in LVGS (R = 0.607, p < 0.001) was observed. ROC curve analyses revealed an AUC of 0.747 (p = 0.02, 95% CI 0.632–0.832) for PWV. A PWV value of 9.2 m/s predicted CTRCD with 94% sensitivity and 73% specificity. AS measurement may be useful for predicting CTRCD in patients with low to medium-risk BC.

**Graphical Abstract:**

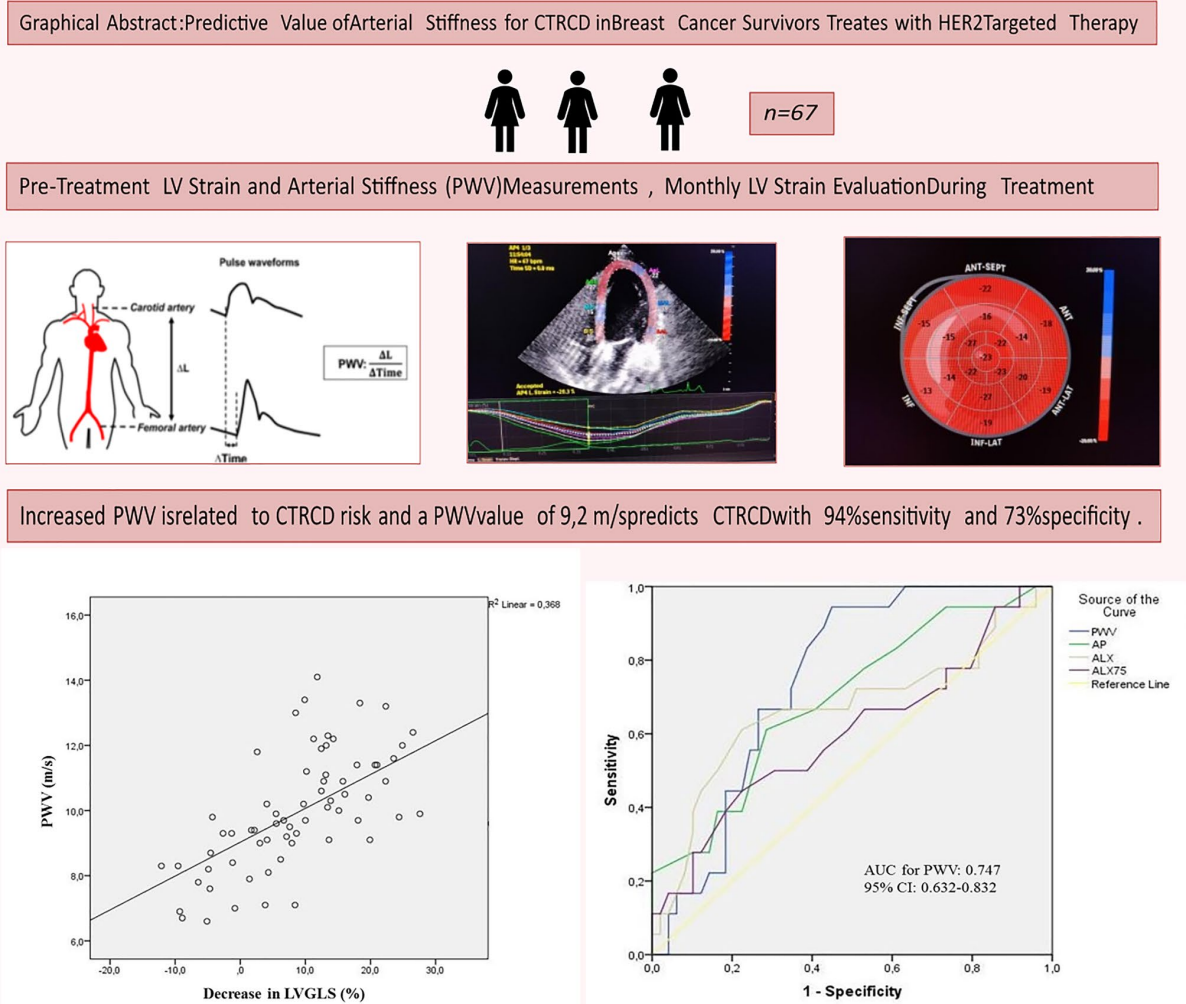

## Introduction

Breast cancer (BC) is the most common cancer in the world. Anthracyclines and taxanes are the main chemotherapeutic agents used in BC. Anti-human epidermal growth factor receptor-2 (HER2) targeted therapies have improved outcomes in patients with HER-2-positive disease [1]. Despite the efficacy of these treatments, cancer therapy-related cardiac dysfunction (CTRCD) risk is high and may limit the benefits and increase cardiovascular risk. Predicting high-risk patients is crucial for intensive cardiovascular screening and preventive strategies. Despite efforts to establish a useful predictive model, there is currently no validated and routinely used one. The weakest aspect of risk scores is that they are not personalized. The influence of a given cardiovascular (CV) risk factor on the development of CV events may be different if it is well controlled or not [2, 3]. Recently, the European Society of Cardiology and the International Cardio-Oncology Society recommended charts for baseline CV risk assessment of anticancer therapies, including anthracyclines and anti-HER2 agents [4]. While risk factors for CTRCD development are well known, existing risk scores are yet insufficient in real-world clinical settings [5]. Suntheralingam et al. [6] evaluated three risk models (Ezaz et al., NSABP-31 cardiac risk scores, and HFA-ICOS trastuzumab Pro-forma) and reported that the performance of the models concerning its discrimination for CTRCD and its calibration with published/suggested incidence was limited, especially for low-risk patients.

Arterial stiffness (AS) is an indicator of the elasticity of the blood vessel wall. It is a non-invasive method of measuring endothelial damage and remodeling and is a predictive marker of subclinical cardiovascular disease. It is a predictor of cardiovascular events and mortality in an asymptomatic population independently from traditional risk factors [7–9]. AS is closely related to most CV risk factors such as age, hypertension (HT), diabetes mellitus (DM), coronary artery disease (CAD), smoking, obesity, and sex [10].

This study aims to evaluate the value of AS in predicting subsequent CTRCD in patients with BC undergoing cardiotoxic treatment. Although many studies in the literature show that AS is impaired in patients receiving chemotherapy, no studies show whether it can be used for risk assessment before treatment.

## Method

**Fig. 1:**
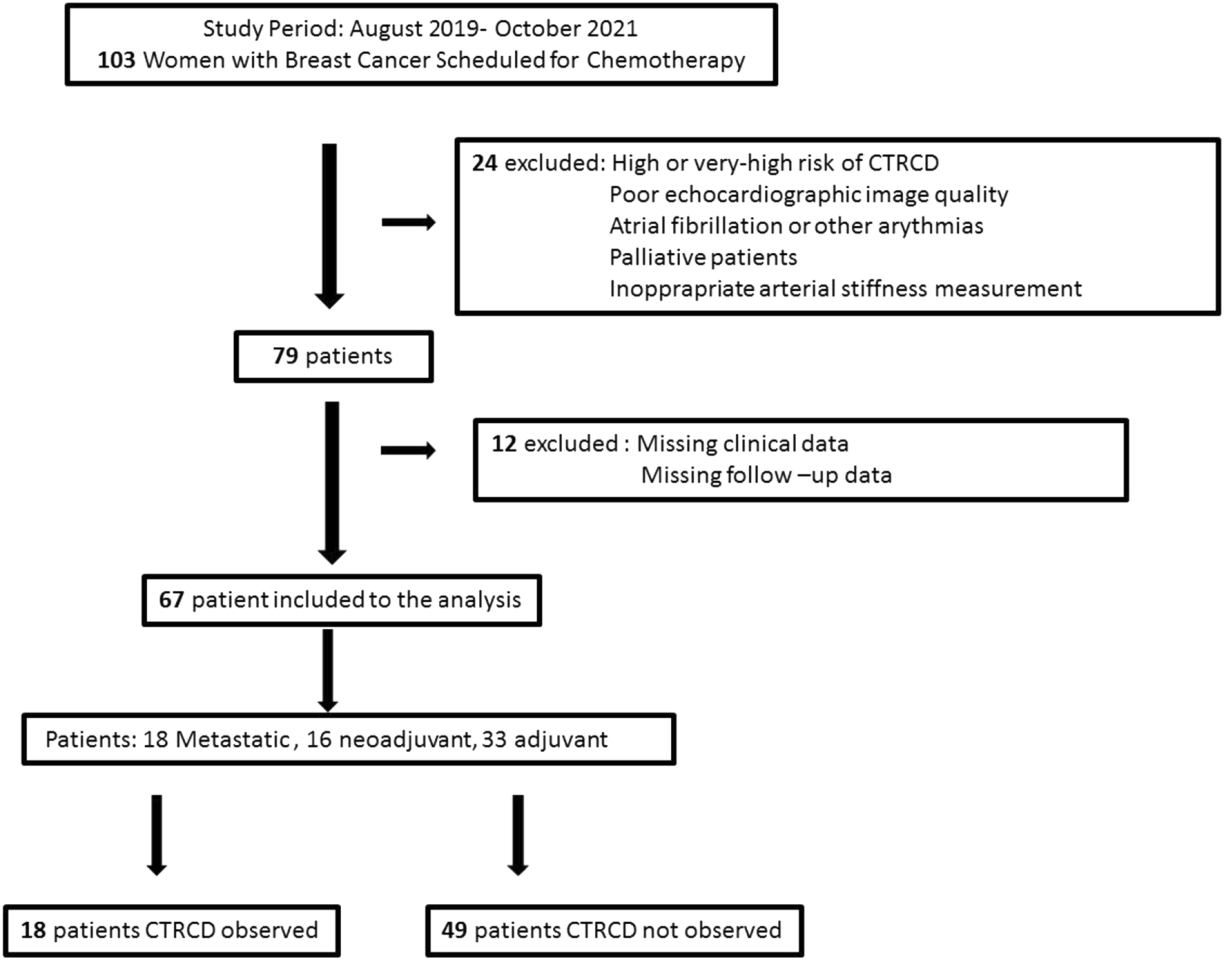
Data collection flowchart

In this prospective cohort study, we included low to medium-risk patients with HER2-positive, stage I to III BC, who would be treated with trastuzumab (± pertuzumab) with or without anthracyclines at the Karadeniz Technical University Hospital between 2019 and 2021 (Fig. 1). Before treatment, all patients' characteristics and baseline risk factors were noted. The exclusion criteria were high-risk patients at baseline according to risk scores (age > 75 years, prior low ejection fraction (< 50%), prior anti-cancer treatment, or radiotherapy), patients who were deemed palliative care, poor echocardiographic image quality, unable to measure AS, patients with atrial fibrillation, or other arrhythmias. All patients received adriamycin/epirubicin and cyclophosphamide and/or paclitaxel/docetaxel with or without trastuzumab ± pertuzumab for first-line therapy. Pertuzumab/trastuzumab alone or together with paclitaxel/docetaxel combination were used as second-line therapy. No patient exceeded the cumulative dose of anthracyclines. In case of disease progression or proven toxicity, the treatment was discontinued. Patients were followed until the completion of the therapy or CTRCD occurred. The patients were analyzed into two groups: CTRCD occurred or not. The study was approved by the hospital ethics research committee, and all patients signed the informed consent.

### Evaluation of CTRCD with Echocardiography

All patients were examined with a Philips Epiq 7 ultrasound system (Philips Health Care, Andover, MA, USA) by a cardiologist at baseline and monthly during follow-up. Left ventricle ejection fraction (LVEF) (with the modified Simpson method), left ventricle diameters and volumes, and left ventricle global longitudinal strain (LVGLS) (with speckle tracking) measurements were obtained. The peak LVGLS was calculated by averaging each of the 16 segments of the left ventricular walls in the two, three, and four-chamber apical views (Fig. 1). The primary definition of CTRCD according to the European Society of Cardiology (ESC) was categorized as severe; if a new LVEF reduction to < 40%, moderate; if a new LVEF reduction by ≥ 10 percentage points to an LVEF of 40–49% or a new LVEF reduction by < 10 percentage points to an LVEF of 40–49% and either a new relative decline in LVGLS by 15% from baseline and mild; if LVEF ≥ 50% and a new relative decline in GLS by 15% from baseline and/or a new rise in cardiac biomarkers [1]. Baseline and post-treatment Hs-Troponin and pro-BNP levels were also evaluated for diagnosis.

### Evaluation of AS

Arterial stiffness was non-invasively assessed with applanation tonometry using the SphygmoCor system (AtCor Medical Pty Ltd, West Ryde, Sydney, Australia). The main index of AS is carotid-femoral pulse wave velocity (PWV). The measurement was performed after 15 min of rest in the supine position. The distances were measured from the carotid pulse site to the femoral artery pulse site. The surface distance between the carotid and femoral sites of measurement may not be correct in obese patients. Therefore, the distance measured in obese patients was fixed by multiplying by 0.8 [11]. The measurements were obtained with simultaneous ECG recordings. Arterial pressure waveforms were recorded at the carotid, radial, and femoral arteries by a tonometer. The software automatically processes the recorded pressure waveforms and computes the PWV, augmentation index (AIx), augmentation pressure (AP), and AIx@75 (Because AIx is influenced by heart rate, an index normalized for a heart rate of 75 bpm (AIx@75) is used). All measurements were performed by an experienced investigator blinded to the patient's information. Only high-quality recordings (mean quality index > 80%) were included in the analysis [12–14].

### Statistical Analysis

Statistical analyses were performed using IBM SPSS V23. Normal distribution was evaluated by the Kolmogorov–Smirnov and Shapiro–Wilk tests. Chi-square and Fisher's Exact tests were used to compare categorical variables according to groups. An Independent two-sample t-test was used for comparing continuous variables. C-reactive protein values were non-normally distributed and were analyzed with the Mann–Whitney U test. Continuous variables are expressed as mean ± standard deviation or median (Q1-Q3), whereas categorical variables are expressed as frequencies and percentages. Pre-treatment and post-treatment LVEF and LVGS values were compared with Paired samples T-Test. Binary logistic regression analysis (Enter model) was used to examine the risk factors affecting CTRCD. The established model was significant with the tests regarding the model fit (χ^2^ = 8.415; p = 0.015). PWV cut-off points were generated to calculate sensitivity and specificity for CTRCD with receiver operating characteristic (ROC) curves. Pearson's correlation analyses were used for evaluating the relationship between PWV and the decreased percent of LVGS. The significance level was presented as p < 0.050.

## Results

**Fig. 2:**
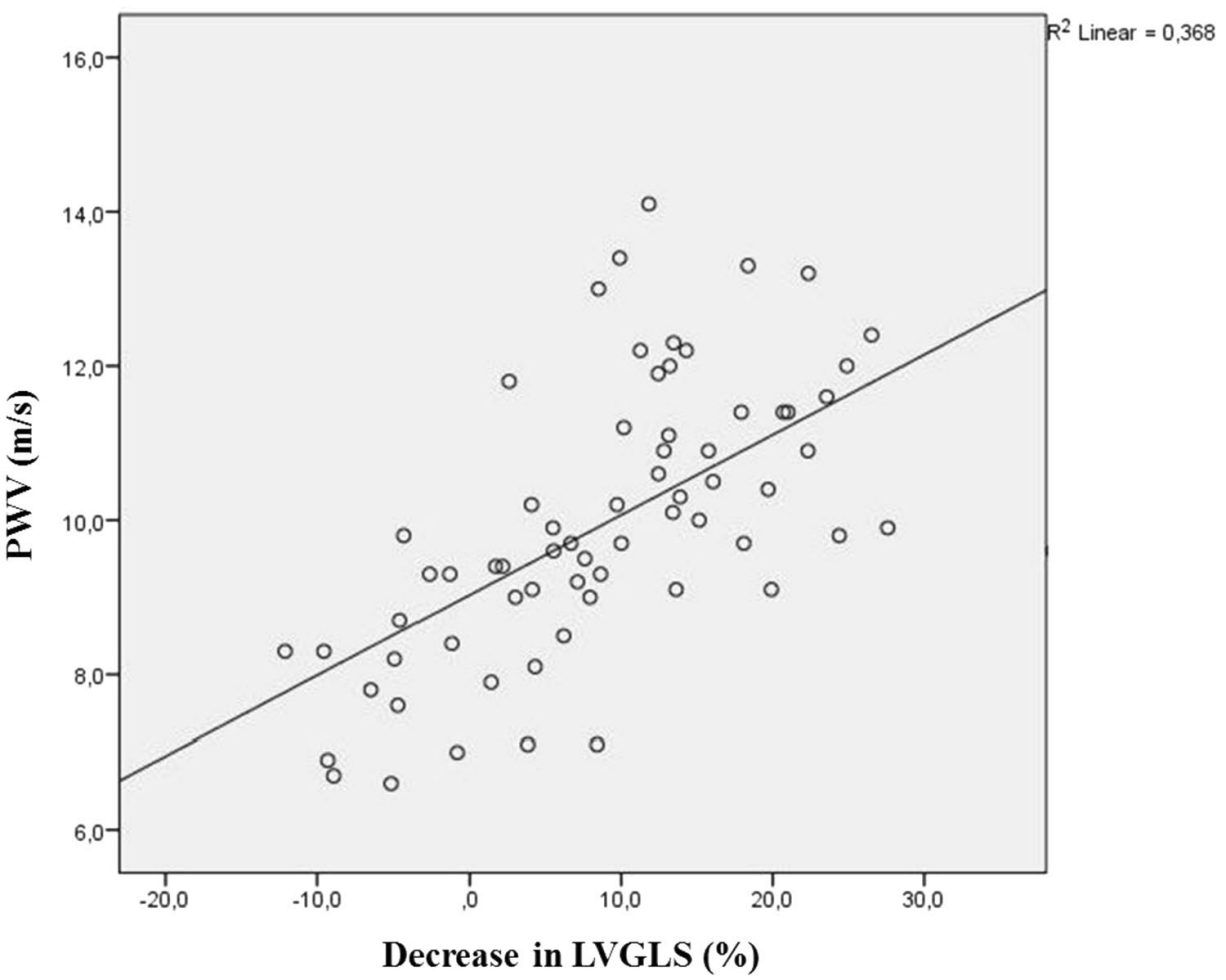
The regression graph of the PWV and percent decrease in LVGLS

**Fig. 3:**
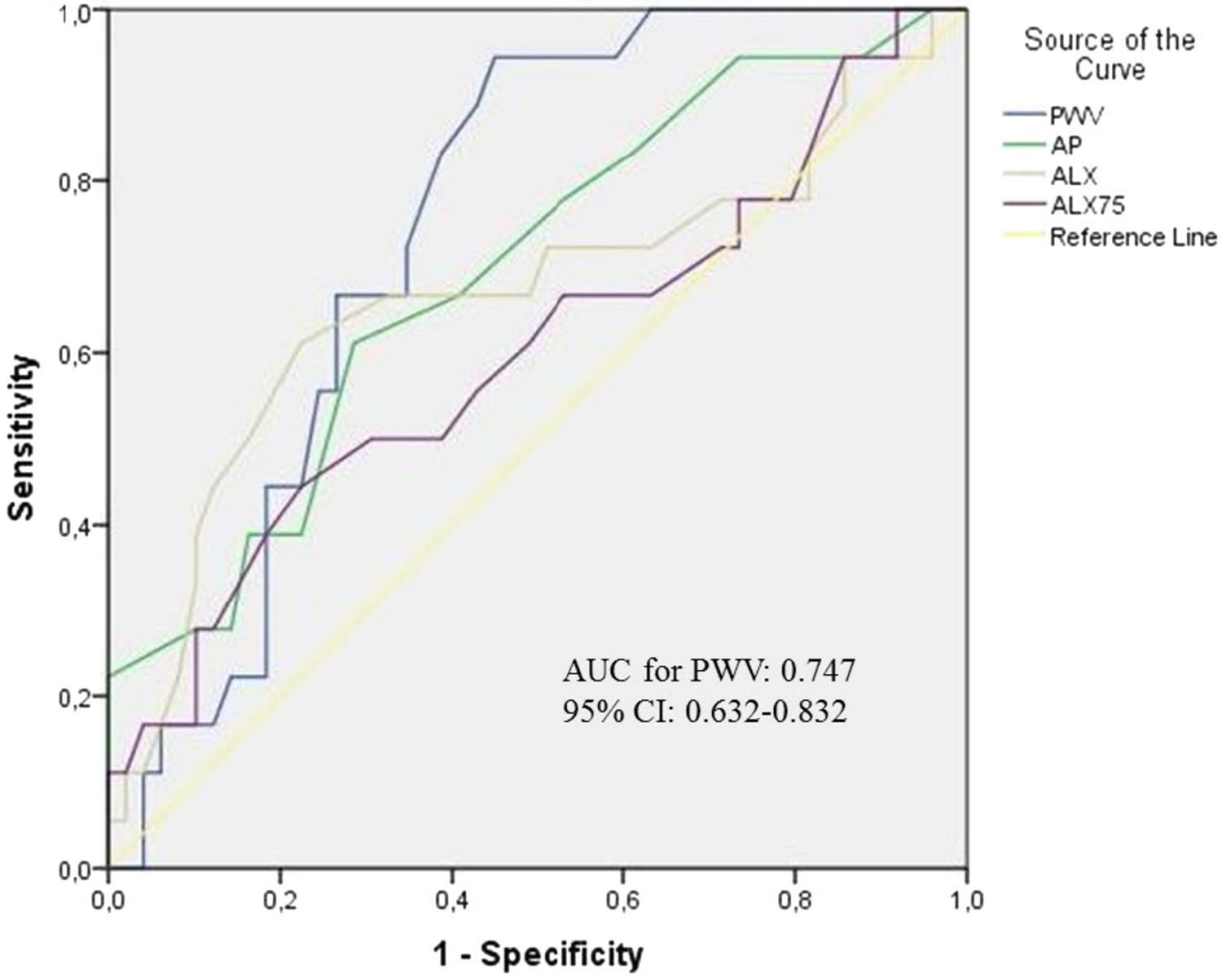
Receiver operative characteristic curve analysis of predictive variables of arterial stiffness parameters

A total of 67 patients were included in the analyses. The mean age of the study population was 54.9 ± 11 years. HT and DM were present in 41% (n = 28) and 20% (n = 14) of the patients, respectively. Baseline characteristics and comparisons of the study groups are given in Table 1) Pre-treatment and post-treatment LVEF and LVGS values were compared with paired samples T-Test and both were statistically significant (p < 0.001). Patients did not demonstrate LVEF reduction, but 18 patients (26.8%) developed mild CTRCD with a reduction in GLS of more than 15% (up to 38.5). When the groups were compared regarding clinical risk factors, only age and GFR were significantly different (p = 0.001, p = 0.036, respectively). Left ventricle hypertrophy and diastolic dysfunction were more frequent in patients with CTRCD (p = 0.016 and p = 0.015, respectively). PWV, AIx, and AP as AS parameters were significantly higher in patients with CTRCD, but Alx@75 was not (p = 0.005, p = 0.034, p = 0.008, p = 0.077 respectively). Pearson's correlation analyses showed a positive correlation between PWV and a decrease percent in LVGS (R = 0.607, p < 0.001) (Fig. 2). Binary multiple regression analyses revealed a significant relation between CTRCD and baseline LVGS and PWV (OR: 1.1, p = 0.031, 95% CI 0.629–1.248 and OR: 1.2, p = 0.022, 95% CI 0.535–2.849, respectively) (Table 2). ROC curve analyses revealed an AUC of 0.747 (p = 0.02, 95% CI 0.632–0.832) for PWV (Fig. 3). A PWV value of 9.2 m/s predicted CTRCD with 94% sensitivity and 73% specificity [Positive predictive value of 67.0% (95% CI 0.56 to 0.77) and a negative predictive value of 95.2% (95% CI 0.76 to 0.97)].

**Table 1:** Baseline and post chemotherapy characteristics

	All patients n = 67	Patients with CTRCD n = 18	Patients without CTRCD n = 49	p value
*Baseline clinical features*				
Age (years)	54.9 ± 11.5	62.2 ± 10.4	52.2 ± 10.8	**0.001**
Hypertension (n, %)	28 (41.8)	11 (61.1)	17 (34.6)	0.052
Diabetes mellitus (n, %)	14 (20.9)	4 (22)	10 (20)	0.558
Coronary artery disease (n, %)	13 (19.4)	5 (27)	8 (16)	0.223
Body mass index (kg/m^2^)	29.6 ± 4.9	28.9 ± 4.8	29.9 ± 4.9	0.475
Glomerular filtration rate	101.6 ± 15.3	95.2 ± 16.9	104.0 ± 14.1	**0.036**
Smoking (n, %)	12 (17.9)	1 (5)	11 (22)	0.103
Hemoglobin (mg/dL)	12.9 ± 1.1	13.1 ± 1.0	12.9 ± 1.2	0.579
Low density lipoprotein (mg/dl)	131.3 ± 23.2	137.0 ± 23.4	129.1 ± 23.0	0.222
Leukocyte (× 10^6^/mm^3^)	7.2 ± 1.8	6.9 ± 1.6	7.3 ± 1.9	0.482
C-reactive protein (ng/dL)	0.62 (0.3–12.3)	4.05 (0.4–10.3)	4.12 (0.3–11.4)	0.172
Hs-Troponin-T (ng/L)	11.3 ± 1.8	10.5 ± 2.0	12.1 ± 3.3	0.501
Pro-BNP (ng/L)	111.5 ± 20.7	126.2 ± 18	102.9 ± 26.1	0.648
*Baseline echocardiographic measurements*				
LVEF (%)	63.1 ± 3.1	62.6 ± 2.4	63.2 ± 3.4	0.485
LVESD (mm)	29.0 ± 3.7	29.5 ± 3.9	28.8 ± 3.6	0.512
LVEDD (mm)	45.2 ± 3.2	45.7 ± 4.2	45.0 ± 3.4	0.494
LVDV (mL)	87.8 ± 9.2	88.9 ± 12	87.4 ± 7.9	0.560
LVH (n, %)	22(32.8)	10(55)	12(24)	**0.016**
LVDD (n, %)	32(47.8)	13(72)	19(38)	**0.015**
LVGS (%)	− 21.1 ± 2.4	− 21.3 ± 2.1	− 21.0 ± 2.5	0.653
*Arterial stiffness measurements*				
PWV (m/s)	9.97 ± 1.7	10.97 ± 1.2	9.61 ± 1.8	**0.005**
Aıx	27.7 ± 8.7	31.4 ± 11.0	26.3 ± 7.4	**0.034**
AP	10.0 ± 4.5	12.4 ± 4.8	9.1 ± 4.1	**0.008**
AIx@75	29.2 ± 8.2	32.2 ± 9.8	28.2 ± 7.3	0.077
Heart rate(per/min)	78.3 ± 10.4	76.7 ± 11.2	78.9 ± 10.3	0.447
*Post chemotherapy echocardiographic measurements*				
LVEF (%)	61.7 ± 3.0	60.1 ± 1.3	62.3 ± 3.2	**0.007**
LVESD (mm)	29.3 ± 3.4	28.9 ± 3.7	29.4 ± 3.3	0.614
LVEDD (mm)	45.2 ± 3.6	45.7 ± 4.2	45.0 ± 3.4	0.425
LVDV (mL)	87.8 ± 8.8	89.9 ± 11.9	87.0 ± 7.3	0.232
LVH (n, %)	22 (32.8)	10 (55)	12 (24)	**0.032**
LVDD (n, %)	40 (59.7)	15 (83)	23 (46)	**0.041**
LVGS (%)	− 19.3 ± 3.2	− 16.7 ± 1.9	− 20.2 ± 3.1	**< 0.001**

Post chemotherapy				
Cardiac markers*	n = 20	n = 8	n = 12	p value
Hs-Troponin-T (ng/L)	22.8 ± 10.6	35.7 ± 8.4	15.7 ± 4.6	**–**
Pro-BNP (ng/L)	196.3 ± 58.4	226.3 ± 48.4	146.6 ± 33.4	**–**

Bold values indicate significance of p value (p < 0.05)

Frequency (%), mean ± standart deviation, median (Q1–Q3)

*LVGLS* left ventricle global longitudinal strain, *LVEDV* left ventricular end-diastolic volume, *LVEF* left ventricular ejection fraction, *LVESD* left ventricular end-systolic diameter, *LVEDD* left ventricular end-diastolic diameter, *LVH* left ventricle hypertrophy, *LVDD* left ventricle diastolic dysfunction, *PWV* pulse wave velocity, *Alx* augmentation index, *AP* augmentation pressure

*Post-chemotherapy cardiac markers were available for 20 patients, eight and twelve patients, respectively with CTRCD, and without CTRCD

**Table 2:** Binary logistic regression analysis of cardiotoxicity predictors

	OR	B	p value	95% confidence İnterval	
Age	1.102	0.097	0.245	0.936	1.298
Hypertension	0.165	− 1.802	0.175	0.012	2.226
Diabetes mellitus	0.254	− 1.370	0.220	0.028	2.272
Coronary artery disease	1.567	0.449	0.625	0.259	9.484
Smoking	0.093	− 2.371	0.122	0.005	1.880
Glomerular filtration rate	0.968	− 0.033	0.380	0.900	1.041
Baseline LVEF	0.855	− 0.157	0.200	0.672	1.086
LVDD	0.406	− 0.901	0.066	0.040	4.108
LVH	1.575	2.679	0.057	0.923	2.209
Baseline LVGS	1.18	− 0.583	**0.031**	0.629	1.248
PWV	1.234	0.210	**0.022**	0.535	2.849
AP	1.418	0.350	0.078	0.962	2.091
ALX	0.862	− 0.149	0.081	0.658	1.129
ALX@75	1.045	0.044	0.649	0.866	1.260

Bold values indicate significance of p value (p < 0.05)

Goodness-of-fit

R = 0.866|R2 = 0.750|Adjusted R2 = 0.695|SE = 0.982

*LVGLS* left ventricle global longitudinal strain, *LVEF* left ventricular ejection fraction, *LVH* left ventricle hypertrophy, *LVDD* left ventricle diastolic dysfunction, *PWV* pulse wave velocity, *Alx* augmentation index, *AP* augmentation pressure

The dependent variable is the development of CTRCD

## Discussion

In our prospective cohort of patients with BC treated with anthracyclines and anti-HER2 agents, we observed that PWV as an AS parameter may predict CTRCD throughout treatment in low and medium-risk patients. Risk prediction is an important concern for several clinical conditions. Determining patient-specific risk mostly remains limited [15]. Clinical risk factors may not pose a similar risk in every patient. It is certain that controlled versus uncontrolled chronic diseases like DM, HT, and CVD will not pose a similar risk among patients. This reduces the power of clinical risk scores. To accurately risk stratify patients for CTRCD, novel strategies are required, like genetic factors, novel imaging measures, and blood biomarkers. AS is an important risk factor and a useful prognostic marker for CV events, including CV diseases, HT, DM, and renovascular diseases [7]. AS also predicts incident events independently of adjustment for CV risk factors in a healthy population [10]. AS reflects the presence of composite end-organ damage and has been shown to have superior prognostic value to measurements of office and ambulatory systolic blood pressures [16]. Most of the traditional risk factors for CTRCD like older age, HT, DM, CAD, prior myocardial infarction, prior cardiotoxic treatment, and radiotherapy are related to increased AS [1]. To the best of our knowledge, this study is the first to demonstrate if AS could predict CTRCD.

The PWV is the gold standard for evaluating AS. PWV is an independent predictor of CV events [17]. The predictive ability is higher in patients with a higher baseline CV risk [18]. We used the carotid-femoral PWV method for measurements with applanation tonometry. Although it is the gold standard method, it has some limitations. The method is not well standardized, and it is experience-dependent. In obese patients, the surface distance between the carotid and femoral sites, which is very important for calculations, may not be adequately measured and may cause erroneous results. Patients with irregular rhythms like atrial fibrillation and frequent early beats are not appropriate candidates for AS measurements. Other techniques like ambulatory AS index, cardio ankle vascular index, and AS measurement by echocardiography and magnetic resonance imaging are shown to be similar [19]. Despite its known prognostic implications, PWV has limited clinical use due to a lack of well-defined cut-offs. Age is a well-described factor related to increased AS. With aging, collagen deposition increases, the mechanical properties of the vascular media change, and maladaptive remodeling occurs [20]. Other risk factors, including HT and DM, also contribute to an increase in AS with age [21]. HT shows a very strong and interdependent relationship with AS [22]. Increased AS preceding the development of overt HT has been demonstrated in population-based studies [23]. Based on clinical outcome data, the 2007 ESC/ESH guidelines recommend a cut-off of 12 m/s for increased AS [24]. But a single threshold also has limitations. For example, age has a dominant effect on PWV, and there have been attempts to establish reference values for various segments of different age groups. In our study population, the mean PWV was 10.97 ± 1.2 m/s and 9.61 ± 1.8 m/s in patients with and without CTRCD, respectively. A PWV value of 9.2 m/s predicted CTRCD with 94% sensitivity and 73% specificity. The population of this study consisted of relatively low-risk patients with a mean age of 55 years and low rates of other CV risk factors. This may explain a relatively low PWV cut-off for CTRCD. These values need to be validated in a large group of the same patient population. CTRCD rate in our study was 26.8% with the definition according to a decline in LVGLS. This rate is similar to those reported in older studies ranging from 18.6 to 32.0% [25, 26]. No CTRCD was observed according to the definition of LVEF decline. This is possibly related to; 1- The small number of patients, 2- Included patients were low to medium-risk patients, 3- Less toxic epirubicin was used instead of doxorubicin, 4- 10% of the patients were treated with dexrazoxane, a cardioprotective agent. There are several limitations to this study. The small sample size was the most important limitation. A large, well-randomized, controlled study evaluating AS as a predictive tool for CTRCD is mandatory. The measurement of PWV by the oscilloscope method requires experience and can have intra and interobserver differences. Since this measurement was made by an experienced operator in this field and due to the Covid-19 pandemic, a second operator measurement was not performed. For the same reason, the diagnosis of cardiotoxicity could not be supported with troponin and natriuretic peptide levels for all patients. Since this study is the first study on this subject, we found it appropriate to measure AS using the gold standard method, the applanation tonometer. This method is indeed difficult to access, requires technical experience, and takes time. However, other validated oscillometric methods measure AS more practically (Mobile-graph, Complior, PulsePen, etc.) [27]. Several studies have shown that the measurement of arterial elastance (Ea) by echocardiography is similar to that obtained by tonometric methods. It can be noninvasively measured as the ratio of end-sistolic pressure (ESP) to stroke volume (SV), an echocardiographic method (Chantler formula) that is more accessible and easy to perform for clinical cardiologists [28, 29]. One of the controversial issues about PWV is the uncertainty of cut-off values. It varies in different clinical situations. More comprehensive studies are needed to determine the PWV threshold value required to predict cardiotoxicity. Lastly, the sample size consisted of low to medium-risk patients. The predictive value of AS could be better demonstrated in high-risk patients.

## Conclusion

Patient-specific risk prediction is very important before cardiotoxic treatment. AS measurement may be a unique risk stratification tool opposed to a "one-size-fits-all" approach in low to medium-risk patients with BC.

## Data Availability

The data that support the findings of this study are available from the corresponding author upon reasonable request.
